# The Evolution of IL6-IL6R-JAK-STAT Signaling Pathway in Metazoan

**DOI:** 10.3390/biology15100753

**Published:** 2026-05-09

**Authors:** Hong Yu, Renle Chang, Houyou Wang, Muchun He, Jiejie Sun, Linsheng Song

**Affiliations:** 1 Liaoning Key Laboratory of Marine Animal Immunology, Dalian Ocean University, Dalian 116023, China; y984664@163.com (H.Y.); renlechang@163.com (R.C.); 15508386010@163.com (H.W.); lshsong@dlou.edu.cn (L.S.); 2 Liaoning Key Laboratory of Marine Animal Immunology & Disease Control, Dalian Ocean University, Dalian 116023, China

**Keywords:** cytokines, cytokine signaling pathway, metazoan, Mollusca, Porifera, innate immunity, aquaculture industry, phylogenetic analysis

## Abstract

Background: The Interleukin-6 (IL6)–Interleukin-6 receptor (IL6R)–Janus kinase (JAK)–signal transducer and activator of transcription (STAT) signaling pathway plays a crucial role in innate immunity by inducing the production of various immune effectors. However, its stepwise evolutionary assembly across metazoans remains incompletely characterized. Methods: This study systematically screened 18 metazoan phyla or subphyla for the presence of IL6 family cytokines, their receptors, JAKs, and STATs. Phylogenetic relationships were constructed using Neighbor-Joining methods, and domain architectures were predicted via SMART. Results: IL6 was found only in vertebrates, whereas IL6R could be traced to Mollusca. JAK and STAT appeared early in Porifera. IL6R, JAK1/2, STAT1 and STAT5A/B first co-existed in Mollusca. The IL-6 co-receptor gp130 was detected in fish, but was not reliably detected in available reptiles’ and birds’ genomes. Conclusions: The IL6-IL6R-JAK-STAT pathway was assembled incrementally: JAK and STAT were first detected in Porifera, IL6R was added in Mollusca to form the IL6R-JAK-STAT module, and finally, most IL6 family cytokines were present in vertebrates (fish). These findings might provide a pre-existing molecular framework that facilitated the subsequent evolution of the complete pathway in vertebrates. Collectively, this study provides a comprehensive evolutionary framework for the stepwise assembly of the IL6-IL6R-JAK-STAT signaling pathway across metazoans.

## Simple Summary

This study traced the evolutionary origins of key components of the IL6-IL6R-JAK-STAT signaling pathway across metazoans. IL6 was only detected in vertebrates, and IL6R could be traced to Mollusca. JAK and STAT appeared early in Porifera. IL6R, JAK1/2, STAT1 and STAT5A/B first co-existed in Mollusca, which marked the earliest presence of the core receptor-kinase–transcription factor module. The IL6 co-receptor gp130 was detected in fish, but was not detected in higher species based on current genomic data, and was re-detected in mammals. Most of the IL-6 family cytokines, along with IL6Rs, JAKs and STATs, were either first detected or re-detected in fish. It revealed how this critical immune pathway was assembled in evolution. These findings provided a framework for understanding the pathway’s origin and diversification in animals.

## 1. Introduction

The IL6R-JAK-STAT signaling pathway is evolutionarily conserved in metazoan (all multicellular animal groups, e.g., poriferans, molluscs, fishes, mammals, and many others) [1]. It can be traced back to Porifera (commonly known as sponges, one of the early-branching animal phyla), as evidenced by the presence of JAK and STAT [2]. The JAK-STAT signaling pathway can be activated by the binding of the IL6 ligands to their receptor complexes, which trigger receptor dimerization and conformational changes essential for JAK phosphorylation and the subsequent recruitment of STAT factors [3]. The IL6R-JAK-STAT signaling pathway plays important roles in various developmental processes, including cellular proliferation, innate immune response, and stem cell development (Table S1) [4,5,6].

The IL6R-JAK-STAT signaling pathway is composed of IL6, IL6R, JAK, and STAT. The IL6 family cytokines include IL6, Interleukin-11 (IL11), Interleukin-27 (IL27), Interleukin-31 (IL31), oncostatin M (OSM), leukemia inhibitory factor (LIF), ciliary neurotrophic factor (CNTF), cardiotrophin 1 (CT-1), and cardiotrophin-like cytokine factor 1 (CLCF1) [7,8]. Their receptors are the dimer of IL6R/Glycoprotein 130 (gp130), IL11R/gp130, IL27R/gp130, IL31R/OSMR, OSMR/gp130, LIFR/gp130, CNTFR/gp130, and CRLF/CNTFR, respectively [9,10,11]. The JAK family contains four cytoplasmic tyrosine kinases (JAK1-3 and TYK2) that associate with the intracellular domains of cytokine receptors [12]. The STAT family consists of the seven intracellular transcription factors (STAT1-6, including STAT5a/5b) [13]. Ligand binding leads to JAK activation and subsequent STAT phosphorylation.

The pathway is conserved in jawed vertebrates [14], but differs markedly in jawless fish and invertebrates [15]. It was first detectable in *Phylum Porifera* [2]. In *Drosophila*, the receptor Domeless (an IL6R homolog) [16], upon binding Upd ligands, activated JAK-STAT signaling [17]. In shrimp, Domeless can bind the IL-like domain of C-type lectin to activate the JAK-STAT signaling pathway [6]. Although JAK and STAT are both present in Mollusca [18] and Porifera [2], IL6R homologs remain poorly understood in lower invertebrates. STAT-like genes (STA-1/2) were also identified from *Caenorhabditis elegans*, while a JAK homolog was absent [19]. Apart from that, there was no information about JAK and STAT homologs in Platyhelminthes [20].

The IL6-IL6R-JAK-STAT signaling pathway plays crucial roles in innate immunity, especially in invertebrates lacking adaptive immunity. In the present study, the main components of this pathway were screened from the genomes of species across metazoan phyla, with the aims of confirming their presence in different species, predicting their evolutionary relationship and structural domains, and providing evidence for a better understanding of the evolution of the IL6-IL6R-JAK-STAT signaling pathway. Unlike previous studies that focused on individual components such as the JAK-STAT module [15] or single receptors [16], the present study provided a simultaneous, multi-component evolutionary tracing of the entire IL6-IL6R-JAK-STAT axis, including ligands, various receptors (IL6R, gp130, IL11R, IL27R, IL31R, OSMR, LIFR, CNTFR, and CRLF1), JAKs, and STATs across 18 metazoan phyla/subphyla. Based on the resulting data, a stepwise assembly model was proposed: JAK and STAT first appeared in Porifera, IL6R was added in Mollusca to form the IL6R-JAK-STAT module, and finally, in vertebrates (fish), most IL6 family cytokines were present. This assembly model represented a conceptual advance for understanding the evolutionary origin of this critical immune pathway. In addition, the domain architectures of all IL6 family receptors were systematically compared across invertebrates and vertebrates. Collectively, these findings provided a novel conceptual framework for understanding how this critical immune signaling pathway was assembled during animal evolution.

## 2. Materials and Methods

### 2.1. The Amino Acid Sequences of IL6-IL6R-JAK-STAT Signaling Pathway

The amino acid sequences of the members in the IL6-IL6R-JAK-STAT signaling pathway were obtained by screening the genomes of different species in metazoan subphyla/phyla from the National Center for Biotechnology Information (NCBI) database. Species were selected based on the availability of high-quality genome assemblies and well-annotated protein sequences in the NCBI database (Table S2), with the aim of covering as many metazoan phyla/subphyla as possible to allow a broad-scale evolutionary comparison. Priority was given to species commonly used as model organisms or with recently published high-coverage genomes. Specifically, the amino acid sequences of well-annotated IL6 family cytokines, IL6R, gp130, JAKs, and STATs from model species including human (*Homo sapiens*), mouse (*Mus musculus*), and zebrafish (*Danio rerio*) were retrieved from the NCBI database to serve as initial query sequences. For lower invertebrates, additional query sequences were obtained from previously reported homologs in closely related species (e.g., IL6R from mollusks and Domeless from arthropods). For each target species (covering mammals, birds, reptiles, fish, cyclostomes, cephalochordates, urochordates, hemichordates, echinoderms, brachiopods, annelids, arthropods, mollusks, nematodes, platyhelminths, coelenterates, and poriferans), tBLASTn and BLASTp (v 2.17.0, https://blast.ncbi.nlm.nih.gov/Blast.cgi, accessed on 5 May 2026) searches were performed against the NCBI Genome, RefSeq, and Protein databases. The BLAST search parameters were set as follows: E-value ≤ 1 × 10^−5^, sequence identity ≥ 30%.

### 2.2. The Evolutionary Analysis of IL6-IL6R-JAK-STAT Signaling Pathway

The amino acid sequences of IL6, IL6R, IL11, IL27, IL31, OSM, LIF, CNTF, CT-1, domeless, gp130, IL31R, LIFR, OSMR, IL11R, IL27R, CNTFR, CLCF1, CRLF, JAK1/2/3, TYK2, STAT1/2/3/4/5/6 from the major metazoan phyla were obtained from the NCBI database. The phylogenetic tree was constructed using the MEGA 7.0 software as in the previously studied methodology [21]. Multiple sequence alignment was performed using Clustal W (https://www.genome.jp/tools-bin/clustalw, accessed on 5 May 2026) with default parameters, followed by manual trimming of ambiguous regions. Phylogenetic trees were constructed with the Neighbor-Joining (NJ) method [22] in MEGA 7.0 (Temple University and University of Pennsylvania, Philadelphia, PA, USA). Evolutionary distances were computed using the p-distance model (pairwise deletion of gaps). Node support was evaluated by bootstrap analysis with 1000 replicates [23]. Under these sequences of high sequence divergence and compositional bias, the NJ method with p-distance was shown to be more reliable than maximum likelihood or Bayesian methods [24], which made it an appropriate choice for this broad-scale evolutionary survey.

### 2.3. The Structural Domains Analysis of IL6-IL6R-JAK-STAT Signaling Pathway

Following the retrieval of amino acid sequences from the NCBI database, the domain structures of IL6R, Domeless, gp130, IL31R, LIFR, OSMR, IL11R, IL27R, CNTFR, JAK1/2/3, TYK2, and STAT1/2/3/4/5/6 across major metazoan phyla were predicted using the Simple Modular Architecture Research Tool (SMART) (https://smart.embl.de/).

### 2.4. The Multiple Sequence Alignment of IL6 Family Members

The amino acid sequences of IL6R and gp130 were analyzed by multiple sequence alignment generated by MEGA 7.0 and GeneDoc (Pittsburgh, PA, USA) software (v2.7, https://genedoc.software.informer.com/, accessed on 5 May 2026). The sequence alignment of IL11R, IL27R, IL31R, OSMR, LIFR, CNTFR and CRLF1 was conducted using the same methods above.

### 2.5. The Evolutionary Tree of Metazoa

All members of the IL6-IL6R-JAK-STAT signaling pathway in this study were screened from the genomes of the following species: mammal, bird, reptile, fish, Cyclostoma, Cephochordata, Urochorda, Hemichordata, Echinodermata, Brachiopoda, Annelida, Arthropoda, Mollusca, Nematoda, Platyhelminthes, Coelenterata, and Porifera.

## 3. Results

### 3.1. Sequential Evolution Analysis and Structural Domain Prediction of IL6 Family Cytokines and Their Receptors

**Figure 1:**
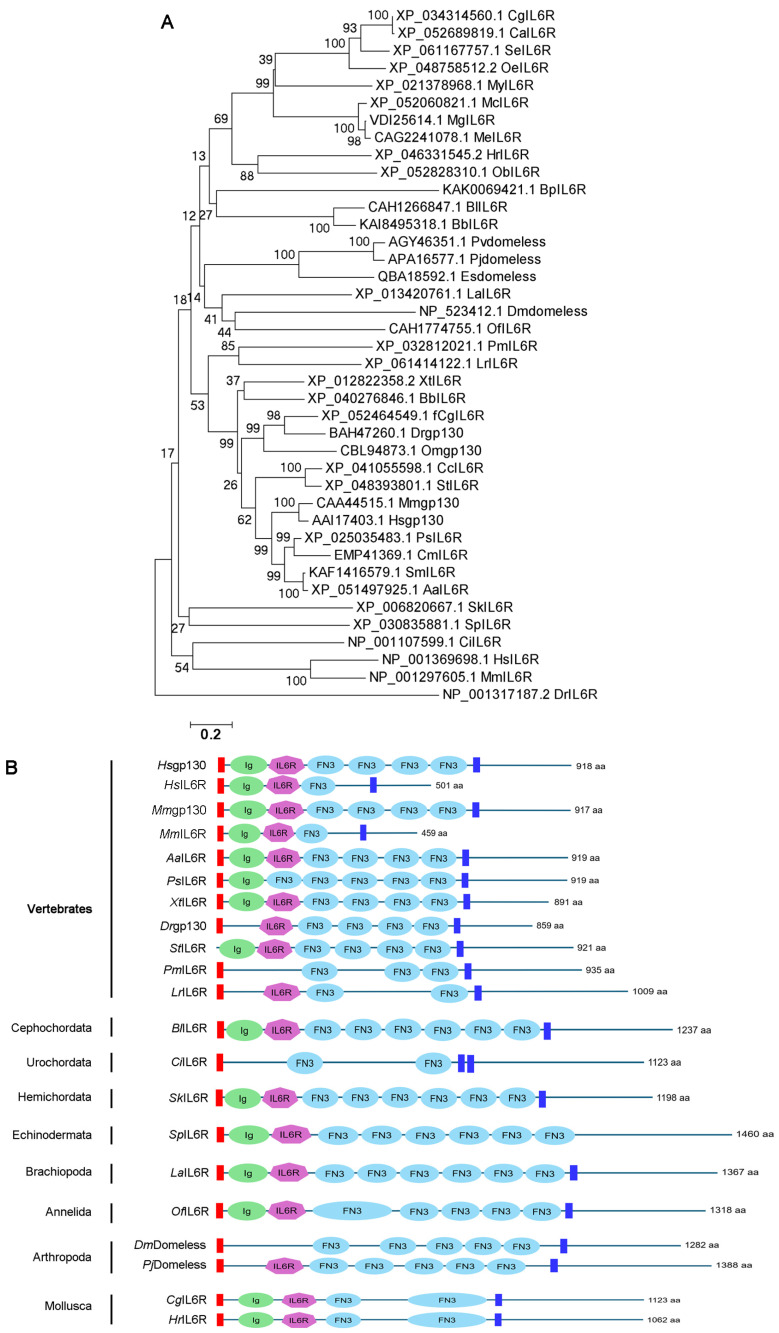
The evolutionary tree and structural domains of IL6Rs and gp130s: (**A**). The evolutionary tree of IL6Rs and gp130s in species of metazoan phyla. (**B**). The structural domains of IL6Rs and gp130s in species of metazoan phyla.

Through multi-sequence alignment of the amino acid sequences of IL-6 family cytokines (IL6, IL11, IL27, IL31, CNTF, CLCF-1, and CT-1), it was found that the IL-6 family cytokines were not conserved across vertebrates (Figure S1). In the evolutionary tree of IL6Rs and gp130s from metazoan phyla, IL6Rs from humans, mice and fish had a closer evolutionary relationship with those from *Ciona intestinalis* of Urochorda, *Saccoglossus kowalevskii* of Hemichordata, and *Strongylocentrotus purpuratus* of Echinodermata. gp130s from humans, mice and fish had a closer evolutionary relationship with IL6Rs from birds, reptiles, Amphibians, fish and Cyclostoma. Domeless from Arthropoda and IL6Rs from Mollusca were clustered together, respectively (Figure 1A). The structural domains of IL6R and gp130 were very similar, and they normally had a signal peptide, an Ig domain, an IL6R domain, three to four FN3 domains, a transmembrane region, and a cytoplasmic tail (Figure 1B).

**Figure 2:**
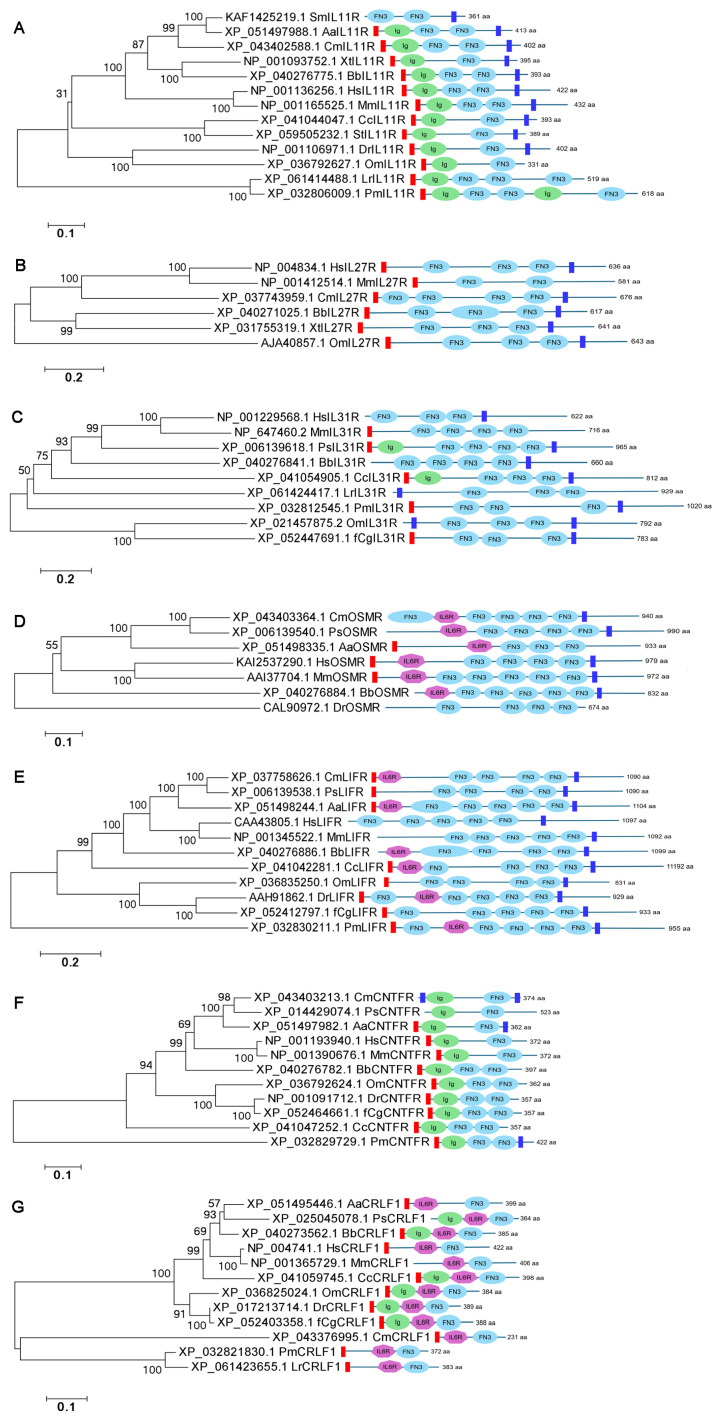
The evolutionary tree and structural domains of the receptors of IL6 family members. The evolutionary tree and structural domains of IL11Rs (**A**), IL27Rs (**B**), IL31Rs (**C**), OSMRs (**D**), LIFRs (**E**), CNTFRs (**F**) and CRLF1s (**G**) in species of metazoan phyla. The bootstrap values lower than 30 were omitted in the tree.

According to the evolutionary trees of IL11Rs, IL27Rs, IL31Rs, OSMRs, LIFRs, CNTFRs and CRLF1s, their relationships were basically consistent with the evolution of the species, respectively (Figure 2A–G). Their structural domains included Ig domain, IL6R domain, FN3 domain, and/or TM domain. Among them, the IL27Rs, OSMRs and LIFRs of all species in the phylogenetic tree lacked the Ig domain. Meanwhile, only OSMRs, LIFRs and CRLF1s contained the IL16R domain (Figure 2A–G).

**Figure 3:**
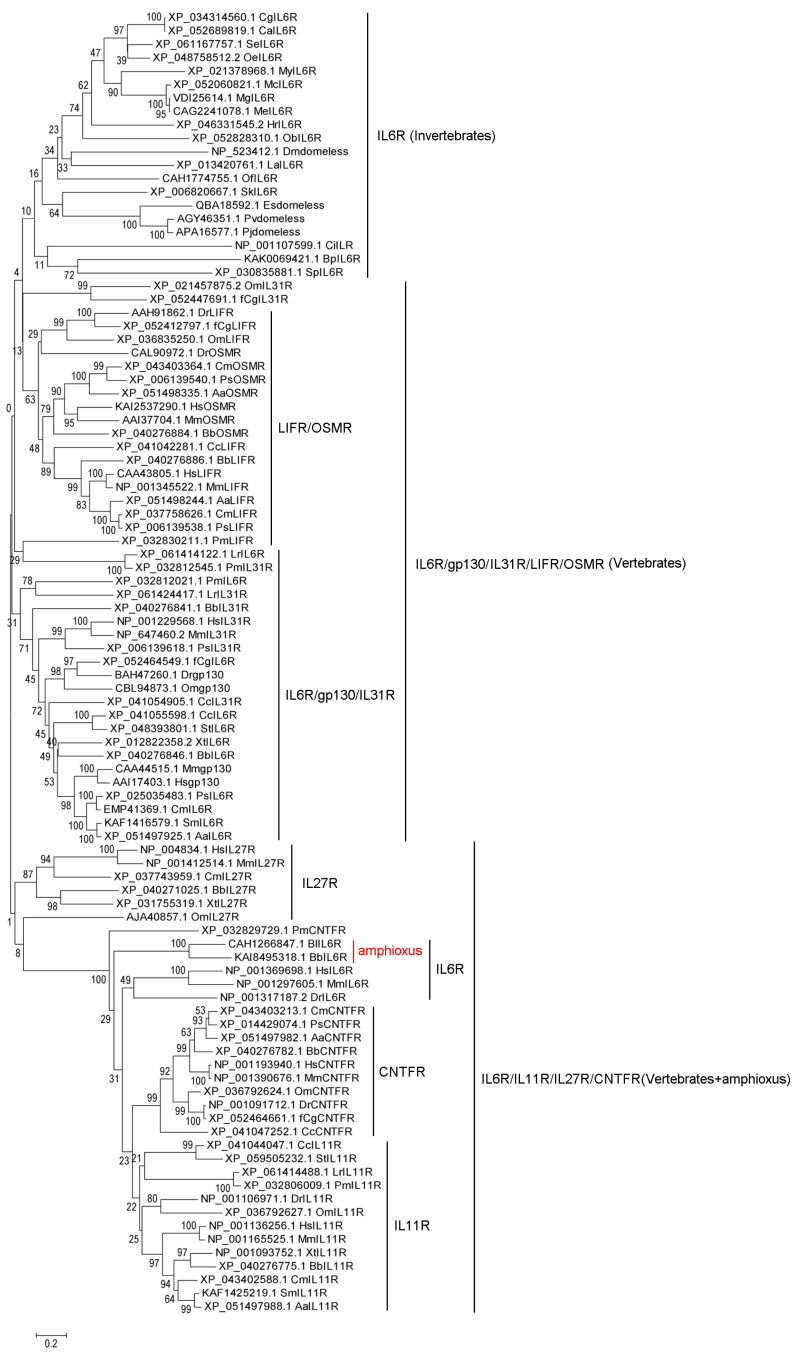
The phylogenetic tree analysis of the receptors of IL6 family members. The phylogenetic tree analysis of IL16Rs, gp130s, IL31Rs, LIFRs, OSMRs, IL27Rs, IL11Rs, and CNTFRs in species of metazoan phyla.

The evolutionary tree was conducted to analyze the evolutionary relationship of the whole receptors of IL6 family cytokines in metazoan phyla. There were three branches of the receptors of IL6 family cytokines in the evolutionary tree. The IL6Rs from invertebrates (except for Amphioxus) clustered together. There were no clearly sub-branches for the receptors from vertebrates. IL6Rs, gp130s, IL31Rs, LIFRs and OSMRs clustered together, and the remaining IL6Rs, IL11Rs, IL27Rs, and CNTFRs clustered together (Figure 3). Among them, LIFRs and OSMRs clustered together, while gp130s, IL31Rs and some IL6Rs clustered together, indicating that LIFRs had a closer evolutionary relationship with OSMRs, and that IL6R and gp130s had a closer evolutionary relationship with IL31Rs and CNTFRs. Meanwhile, IL27Rs, IL11Rs and CNTFRs clustered into a separate clade, respectively (Figure 3).

### 3.2. Sequential Evolution Analysis and Structural Domain Prediction of JAKs

**Figure 4:**
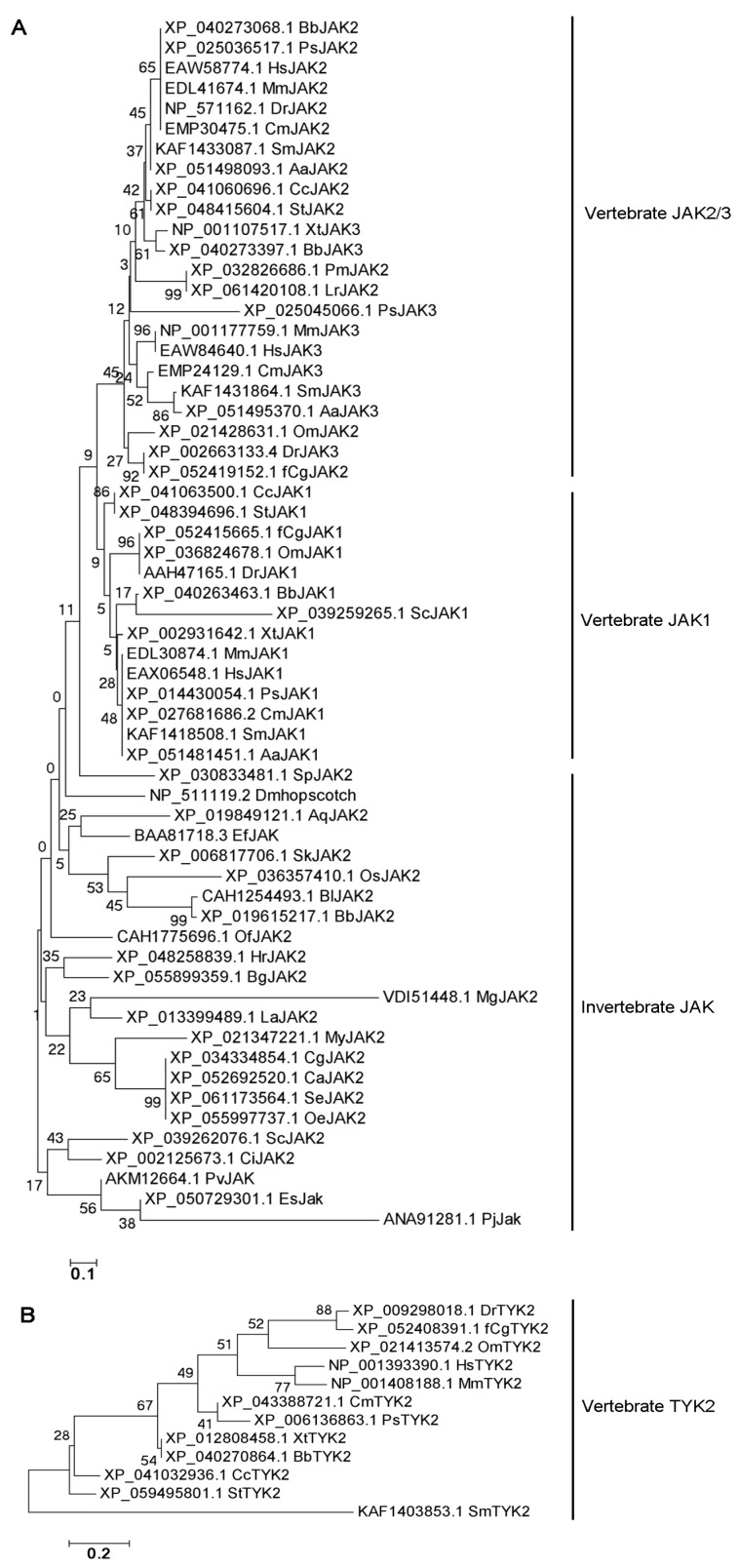
The evolutionary tree of JAKs: (**A**). The evolutionary tree of JAKs in species of metazoan phyla. (**B**). The evolutionary tree of TYK2s in species of metazoan phyla.

**Figure 5:**
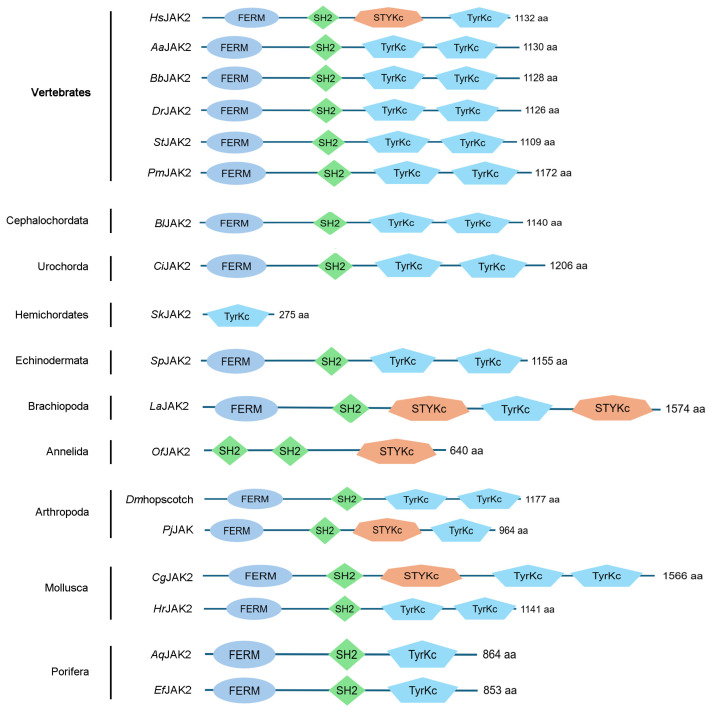
The structural domains of JAKs in multi-species.

According to the evolutionary tree of JAK1s, JAK2s and JAK3s, there were clearly three branches (vertebrate JAK1, vertebrate JAK2/3, and invertebrate JAKs). JAKs in invertebrates had a closer evolutionary relationship with JAK1s in vertebrates (Figure 4A). In the evolutionary tree of TYK2s in vertebrates, TYK2s from humans and mice clustered together with the TYK2s from jawed fishes (Figure 4B). JAK was normally composed of a FERM domain, SH2 domain, STYKc-TyrKc domains or two TyrKc domains, which was conserved in vertebrates. While in some invertebrate phyla, the structural domains were not conserved. Among these, JAK from *Saccoglossus kowalevskii* of Hemichordates contained only the Tyrkc domain, while that from *Owenia fusiformis* of Annelida lacked the FERM domain or the Tyrkc domain (Figure 5).

### 3.3. Sequential Evolution Analysis and Structural Domain Prediction of STATs

**Figure 6:**
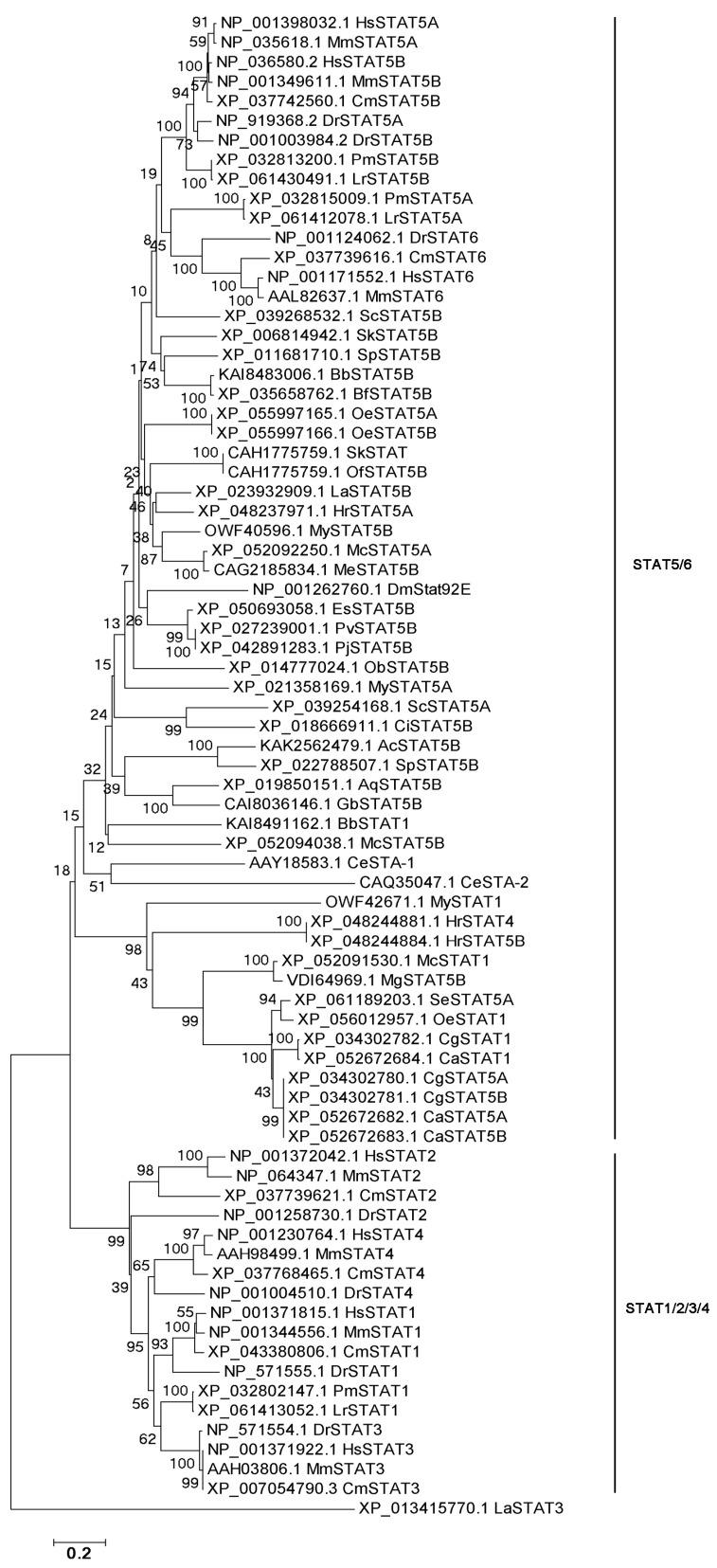
The evolutionary tree of STATs.

**Figure 7:**
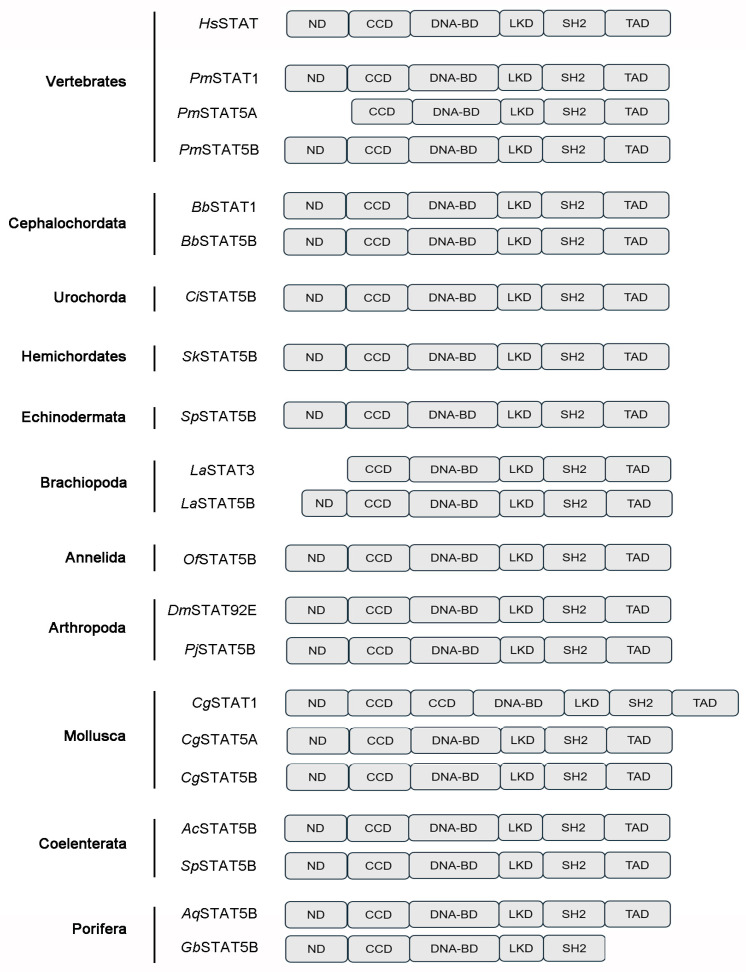
The structural domains of STATs in multi-species.

In the evolutionary tree of STATs, seven STATs from vertebrates were divided into two branches, defined as STAT1/2/3/4 and STAT5/6 (Figure 6). STAT5/6s from vertebrates clustered with STATs from invertebrates, and they all dropped into the STAT5/6 branch (Figure 6). The structural domains of STATs were conserved in different species, and they normally contained a ND, a CCD, DNA-BD, a LKD, a SH2 domain, and a TAD (Figure 7). Among them, STATs from some species lacked the ND domain, such as Lingula anatine of Brachiopoda and Petromyzon marinus of Chordata. Additionally, STAT from Geodia barretti of Porifera lacked the TAD domain.

### 3.4. Evolution of IL6 Family Cytokines, Their Receptors, JAK, and STAT

**Figure 8:**
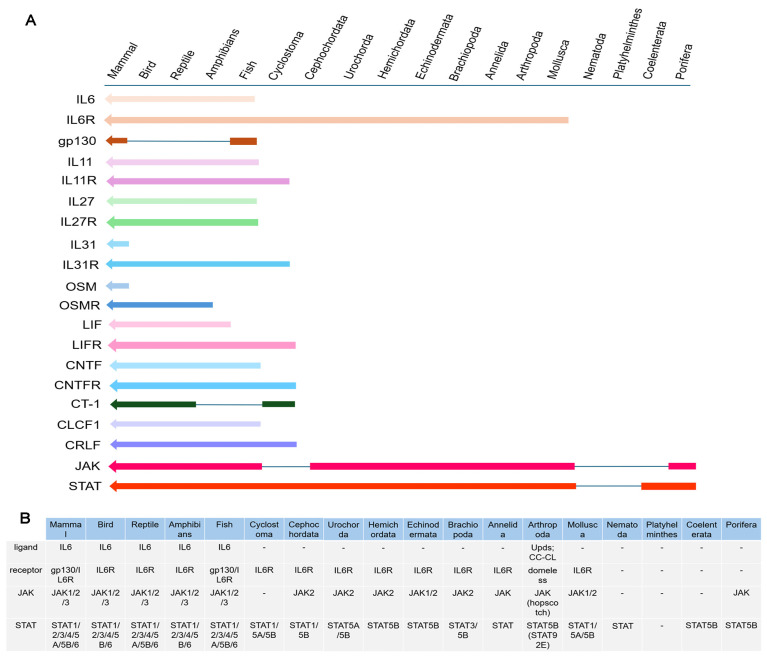
The evolution of IL6 family members and their receptors, as well as JAKs and STATs: (**A**). The evolution of IL6 family members and their receptors, as well as JAKs and STATs, in species of metazoan phyla. (**B**). The presence of IL6s, IL6Rs, gp130s, JAKs and STATs in species of metazoan phyla.

The IL-6 family cytokines were only present in vertebrates. Among IL-6 family cytokines, IL6, IL11, IL27, CNTF, and CLCF1 were detected in fish and higher vertebrates; LIF was detected in amphibians and higher vertebrates. IL31 and OSM were found exclusively in mammals, and CT-1 was identified in cyclostomes and higher vertebrates, with the exception of amphibians and fish (Figure 8A). The receptors of IL6 family cytokines, except for IL6R, were only present in vertebrates. IL11R, IL31R, LIFR, CNTFR and CRLF all first appeared in Cyclostoma. Both gp130 and IL27R first emerged in fish. However, gp130 was not reliably detected in available reptile and bird genomes, and was re-detected in mammals. IL6R could be traced back to Mollusca. JAK and STAT were present early in Porifera. JAK was detected in Coelenterata, Platyhelminthes, Nematoda, and Cyclostoma. Based on current genomic data, STAT was not detected in Platyhelminthes and Nematoda. IL6R, JAK and STAT first co-existed in Mollusca (Figure 8A). IL6 with the IL6 domain was only present in jawed vertebrates (Figure 8A). Although no IL6 had been found in invertebrates, the ligand Upd1/2/3 had been identified in insects of Arthropoda, and an IL-CTL had also been found in Cephalocarida (Figure 8B).

## 4. Discussion

The IL6-IL6R-JAK-STAT signaling pathway plays a significant role in regulating the inflammatory and immune responses [25,26]. IL6 as a cytokine binds with IL6R to induce the dimer of IL6R, which then recruits JAK and STAT. JAK is located on the IL6R to phosphorylate STAT, leading to the dimer of STAT [8,27]. The dimer of STAT eventually translocates from the cytoplasm into the nucleus to regulate the transcription of genes, such as inflammatory cytokines [28] and antibacterial peptides [6]. In the present study, the existence of main components of IL6-IL6R-JAK-STAT signaling pathway, their evolutionary relationship and structural domains were analyzed in different species of metazoan phyla.

The IL6 family cytokines include IL6, IL11, IL27, IL31, OSM, LIF, CNTF, CT-1, and CLCF1. Membership of this cytokine family is defined by the usage of common β-receptor signaling subunits [29]. Members of this family play prominent roles in inflammation, immunity, infectious disease, and even cancer [7,29,30]. In the present study, different IL6 family cytokines were systematically screened from species of different metazoan phyla. They were only found in vertebrates based on the available genome data. Then, IL6 family cytokines were screened from different classes of vertebrates. CT-1 could be traced back to Cyclostoma, while it was not detected in Amphibians and fish. IL6, IL11, IL27, CNTF and CLCF-1 were presented early in fish, and LIF emerged in Amphibians. IL31 and OSM were only found in mammals, while their amino acid sequences were not conserved in different species of vertebrates. These results elucidated that the emergence of IL6 family cytokines represented a continuous process of evolution and natural selection. Though there was no detection of IL6 in invertebrates, the ligand Upd1/2/3 was identified in insecta [31] of Arthropoda, and an IL-CTL was also found in Cephalocarida [6]. Although IL6-like ligands were still not found in most invertebrates, the existence of other types of ligands in the signaling pathway could not be ruled out.

The receptors of IL6 family cytokines are IL6R, gp130, IL11R, IL27R, IL31R, OSMR, LIFR, CNTFR and CRLF, respectively. IL-6 is a 184 amino acid glycosylated protein, which can bind to the IL-6R associated with a second transmembrane protein, gp130 [32,33]. While IL6R and gp130 are both in human [34], mouse [35], and fish [36]. In the present study, the receptors of IL6 family cytokines, except for IL6R, were only detected in vertebrates. IL6R could be traced back to Mollusca. In the evolutionary tree of IL6Rs and gp130s from species of metazoan phyla, IL6Rs from human, mouse and fish had a closer evolutionary relationship with those from species of Urochorda, Hemichordata and Echinodermata, suggesting that IL6Rs from humans, mice and fish might have evolved from those of Urochorda, Hemichordata, and Echinodermata. gp130s from humans, mice and fish had a closer evolutionary relationship with IL6Rs from birds, reptiles, Amphibians, fish, and Cyclostoma. The information from the evolutionary tree of IL6Rs and gp130s suggested that IL6R and gp130 had a closer evolutionary relationship in vertebrates, and they might have come from the same ancestral molecule in invertebrates. IL6Rs and gp130s both co-existed in fish, which might have been due to the gene duplication of IL6R in the deuterostome lineage. And, the expansion of IL-6 family cytokines in fish might have provided the regulatory flexibility required for the sophisticated crosstalk between innate and adaptive immunity. In fish, IL-6, IL-11, and IL-27 are involved in regulating T-cell differentiation [37], B-cell activation [38], and inflammatory balance [39], all of which are essential for adaptive immunity. Domeless from *Drosophila* is a signal-transducing receptor with most similarities to the IL-6 receptor family [16]. In the present study, Domeless from Arthropoda and IL6Rs from Mollusca clustered together, respectively. Domeless clusters with the phylogenetically earliest-diverged IL6R, which provided evidence for the existence of the closest similarity between Domeless and IL6R in terms of their evolutionary relatedness. The evolutionary tree was conducted to analyze the evolutionary relationship of the whole receptors of IL6 family cytokines in metazoan phyla. There were three branches of the receptors of IL6 family cytokines in the evolutionary tree. The IL6Rs from invertebrates (except for Amphioxus) clustered together. There were no clear sub-branches for the receptors from vertebrates. IL6Rs, gp130s, IL31Rs, LIFRs and OSMRs clustered together, and the remaining IL6Rs, IL11Rs, IL27Rs and CNTFRs clustered together. Among these, LIFRs had a closer evolutionary relationship with OSMRs, and IL6Rs and gp130s had a closer evolutionary relationship with IL31Rs and CNTFRs. In murine studies, the two receptors for OSM are formed through the respective associations of OSMR and LIFR with gp130 [40,41]. The closer evolutionary relationship between LIFRs and OSMRs provided a theoretical basis for LIFRs and OSMRs to serve as receptors for OSM, respectively. The structural domains of IL6R and gp130 are very similar and they normally have a signal peptide, an Ig domain, an IL6R domain, FN3 domains, a transmembrane region, and a cytoplasmic tail. However, notable divergence existed across phyla. For example, domeless from *Drosophila* lacked the IL6R domain [16]. This architectural difference suggested a stepwise functional specialization. The acquisition of the IL6R domain in mollusks might have enabled more specific ligand recognition and receptor oligomerization, whereas its undetected presence in arthropods likely reflected a distinct evolutionary trajectory, possibly compensated by alternative ligand-binding modules (e.g., the coiled-coil domain of a C-type lectin) [6]. Whether such alternative ligand-receptor recognition truly leads to signal transduction remains to be tested through ligand-binding and cell-based assays in mollusks and arthropods.

The first enzymatic step in IL-6 signal transduction is the activation of JAK [42]. The interaction of JAK with gp130 was mediated through the FERM domain of JAK [43]. In response to IL-6 binding to the receptor, JAK was activated by autophosphorylation at a double tyrosine motif of the activation loop [44]. Till now, three JAKs (JAK1/2/3) have been identified in mammals [45]. JAK1/2/3 are widely distributed in jawed vertebrates [46]. And in invertebrates, the JAKs of *Drosophila melanogaster* [47] and *Eriocheir sinensis* [48] have been identified and JAKs have not been detected in jawless vertebrates. The absence of JAK in jawless vertebrates and the presence of three JAKs in fishes and even higher species are probably caused by several gene duplications in both jawless and jawed fishes [49]. In the present study, the origin of JAKs was traced back to Porifera, suggesting that JAK might have already existed in Protozoa. This was similar to Nichols (2006), who first reported JAK and STAT in sponges [2]. In addition, Liongue (2012) documented the expansion of JAK-STAT components in teleost fish [15], which was consistent with our finding that most IL6 family cytokines and several receptors first appear or reappear in fish. According to the evolutionary tree of JAKs, JAK2s and JAK3s clustered together, and they had a closer evolutionary relationship than JAK1s in vertebrates. JAKs from invertebrates had a closer evolutionary relationship with JAK1s in vertebrates. The information suggested that in vertebrates, JAK1 is relatively primitive in the JAK family, and JAK2 and JAK3 might have been duplicated from JAK1. And they all came from the same primitive ancestral molecule. JAK is normally composed of a FERM domain, SH2 domain, STYKc-TyrKc domains or two TyrKc domains, which is conserved in Chordata. In contrast, in lower invertebrates, the structural domains showed a great difference. Notably, the JAK homolog of the annelid *O. fusiformis* lacked the FERM domain, which is known to be essential for receptor association [43]. The reason for this phenomenon might be that parts of the gene sequence have been deleted or fused during the evolution of the species. This natural deletion likely impairs its ability to bind cytokine receptors, providing an opportunity to dissect the functional requirement of the FERM domain in JAK-mediated signaling and raising the question of whether these domain losses are compensated by alternative signaling mechanisms awaits experimental validation in representative species.

Activated JAK phosphorylated tyrosine motifs within the cytoplasmic part of gp130 [50]. The five most membrane-distal tyrosine motifs (Y759, Y767, Y814, Y905, and Y915) acted as recruitment sites for signaling components containing SH2 domains [51]. The most prominent proteins recruited to gp130 are the transcription factors of the family of STAT3 and (to a certain extent) STAT1 [52]. The canonical JAK/STAT pathway emphasized binding of STAT1 or STAT3 monomers to one of the four distal tyrosine motifs, tyrosine phosphorylation of the STAT factors, homo- or heterodimerization of tyrosine-phosphorylated STAT1 and/or STAT3 and subsequent translocation of STAT-dimers into the nucleus [15,53]. Till now, seven STATs have been identified from mammals [54]. In the present study, seven STATs first co-existed in fish, while in jawless fishes, there were only STAT1, STAT5A, and STAT5B, suggesting that the gene duplication of the jawed fishes might have led to the presence of seven STATs. In metazoa, STAT5B was detected in Porifera and followed Coelenterata, but was not detected in Platyhelminthes based on current genome data; it was detected again in Mollusca. Concurrently, JAK was detected in Porifera, but was not detected in Coelenterata, Platyhelminthes, and Nematoda, and was detected again in Mollusca. These findings suggested that the JAK-STAT pathway might have originated earliest in Porifera. Although IL6 family cytokines are exclusive to vertebrates, IL6R was first detected in Mollusca. The co-existence of IL6R, JAK and STAT in Mollusca indicated that the IL6R-JAK-STAT signaling pathway might have been present early in Mollusca. In the evolutionary tree of STATs, seven STATs from vertebrates were divided into two branches, defined as STAT1/2/3/4 and STAT5/6. STAT1, STAT2, STAT3 and STAT4 from vertebrates were dropped into the STAT1/2/3/4 branch. STAT5 and 6 from vertebrates clustered with STATs from invertebrates, which were dropped into the STAT5/6 branch. These results indicated that STATs in invertebrates had a closer evolutionary relationship with STAT5/6 in vertebrates. This might imply that STATs in invertebrates and vertebrates share a common origin. During the subsequent evolutionary process, STAT5 and STAT6 in vertebrates have retained more similarities with STATs in invertebrates, while the STAT1/2/3/4 branch has evolved in a different direction, thus giving rise to greater differences. The structural domains of STATs are conserved in different species, and they normally contain a ND, a CCD, a DNA-BD, a LKD, a SH2 domain, and a TAD. From the perspective of species evolution, the domains of STATs are highly conserved. Since STAT first appeared in Porifera, most of its domains have remained stable. However, the STAT protein of the sponge *G. barretti* lacks the TAD, which is required for transcriptional activation [55]. This natural deletion suggests that early STAT molecules may have functioned primarily as DNA-binding adaptors rather than full transcriptional activators. In the species that have been studied, STAT has retained the DNA-binding domain capable of binding to specific DNA sequences, as well as the SH2 domain that participates in protein–protein interactions and thus mediates signal transduction [56,57,58]. This structural stability enables STAT to respond to extracellular signals with similar molecular mechanisms in different species and regulate the transcriptional expression of genes.

The primitive IL6R-JAK-STAT signaling pathway was most likely composed of JAK and STAT. *Monosiga brevicollis* had a STAT-like gene, but no JAK [59]; similar STAT-like genes without a JAK were also detected in *Dictyostelium discoideum* and *Arabidopsis thaliana.* In contrast, both JAK and STAT co-occurred in Porifera and Cnidaria [60]. This suggested that the JAK-STAT core predates animals, whereas the full IL6R-JAK-STAT pathway with dedicated cytokine receptors like IL6R/gp130 appears to be animal-specific. IL6R was early traced back to Mollusca. The co-occurrence of IL6R, JAK, and STAT in Mollusca represented a key finding in the evolutionary assembly of the IL6-IL6R-JAK-STAT axis. Although JAK and STAT were reported in Porifera (sponges), the presence of a membrane-anchored IL6R together with JAK and STAT had not been documented before in any invertebrate phylum. The molluscan module, therefore, constituted the earliest integrated receptor-kinase–transcription factor unit within this signaling pathway. Consistent with the stepwise assembly model we proposed, the molluscan stage added receptor specificity to a pre-existing kinase-transcription factor core. Notably, the co-occurrence of IL6R, JAK, and STAT in Mollusca in the absence of a canonical IL6 ligand suggested that the ancestral receptor-kinase–transcription factor module might have been activated by alternative ligands, as shown for the IL6R homolog Domeless in shrimp, which was activated by a C-type lectin rather than an IL6-like cytokine [6]. These components were continuously identified during evolution, except that in Cyclostoma, JAK had not been detected, whereas in fish, IL6R, JAK, STAT family members, and the ligand IL6 were all present. Thus, the complete IL6-IL6R-JAK-STAT signaling pathway appeared, which is retained till now. In the present study, through the analysis of the presence, evolutionary relationships, and domains of the main components in the IL6-IL6R-JAK-STAT signaling pathway within the metazoan, across various phyla or subphyla, the origin and evolution of the IL6-IL6R-JAK-STAT signaling pathway were clarified in metazoan phyla, which provided important data support for a systematic understanding of the IL6-IL6R-JAK-STAT signaling pathway in metazoan and its evolutionary patterns.

## 5. Conclusions

In this study, the evolutionary origins and assembly process of the IL6-IL6R-JAK-STAT signaling pathway were systematically traced across major metazoan lineages based on available genome data. A stepwise model for the construction of this critical immune pathway was proposed. JAK and STAT had been detected as early as in Porifera, suggesting that the foundational kinase-transcription factor machinery was already present. The core receptor-kinase–transcription factor module, comprising IL6R, JAK1/2, STAT1, and STAT5A/B, was first detected in Mollusca, marking the earliest appearance of a functionally integrated unit. Subsequently, the pathway underwent substantial expansion and refinement in vertebrates, particularly in fish, where most IL-6 family cytokines and key receptors such as gp130 and IL27R were first identified. Notably, gp130 was detected in fish but had not been detected; it was detected again in mammals, suggesting lineage-specific adaptations. This indicated that the complete vertebrate IL6-IL6R-JAK-STAT signaling pathway was not established in a single event, but rather assembled incrementally over hundreds of millions of years through the sequential recruitment and co-option of pre-existing and newly evolved molecules. Collectively, the results provided a comprehensive evolutionary framework for understanding the origin and diversification of one of the most pivotal innate immune signaling pathways in animals.

Based on the evolutionary hypotheses proposed in this study, multiple experiments can be carried out in the future. For example, ligand-receptor binding assays and cell-based signaling reconstitution experiments could be carried out in representative species such as molluscs and early-diverging fish. In addition, alternative analytical approaches, including maximum likelihood or Bayesian phylogenetics with site-heterogeneous mixture models, may help to further resolve the phylogenetic relationships of key components (e.g., the convergent clustering of molluscan IL6R with arthropod Domeless). Meanwhile, inclusion of genomic data from additional metazoan lineages, such as ctenophores and placozoans, would deepen our understanding of how the IL6-IL6R-JAK-STAT pathway was assembled.

## Data Availability

The data presented in this study may be obtained from the corresponding author or the first author upon request.

## Supplementary Materials

The following supporting information can be downloaded at https://www.mdpi.com/article/10.3390/biology15100753/s1. Figure S1: The sequence alignment of IL6 family members. Table S1: IL-6 family cytokines, their receptor complexes and their major biological roles; Table S2: List of species abbreviations. References [61,62,63,64,65,66,67,68,69,70,71,72,73,74,75,76] are cite in Supplementary Materials.

## Abbreviations

The following abbreviations are used in this manuscript:

IL6	Interleukin-6
IL6R	Interleukin-6 receptor
JAK	Janus kinase
STAT	Signal transducer and activator of transcription
gp130	Glycoprotein 130
IL11	Interleukin-11
IL27	Interleukin-27
IL31	Interleukin-31
OSM	Oncostatin M
LIF	Leukaemia inhibitory factor
CNTF	Ciliary neurotrophic factor
CT-1	Cardiotrophin 1
CLCF1	Cardiotrophin-like cytokine factor 1
CRLF	Cytokine receptor-like factor
TYK2	Tyrosine kinase 2
Ig	Immunoglobulin
FN3	Fibronectin type III
TM	Transmembrane
FERM	Band 4.1 (F), Ezrin (E), Radixin (R), and Moesin (M)
SH2	Src homology 2
STYKc	Serine/Threonine/Tyrosine kinase catalytic domain
TyrKc	Tyrosine kinase catalytic domain
ND	N-terminal domain
CCD	Coiled-coil domain
DNA-BD	DNA-binding domain
LKD	Linker domain
TAD	Transactivation domain
SMART	Simple Modular Architecture Research Tool
MEGA	Molecular Evolutionary Genetics Analysis
Upd	Unpaired
IL-CTL	Interleukin-like C-type lectin
VLR	Variable lymphocyte receptor
