# Supplementary material for: The Evolution of IL6-IL6R-JAK-STAT Signaling Pathway in Metazoan
Source: Biology (Basel). 2026 May 9;15(10):753. doi: 10.3390/biology15100753 (PMC13203501; doi:10.3390/biology15100753)
Supplement: Supplementary file 1 [file biology-15-00753-s001.zip › biology-4253025-supplementary.pdf]

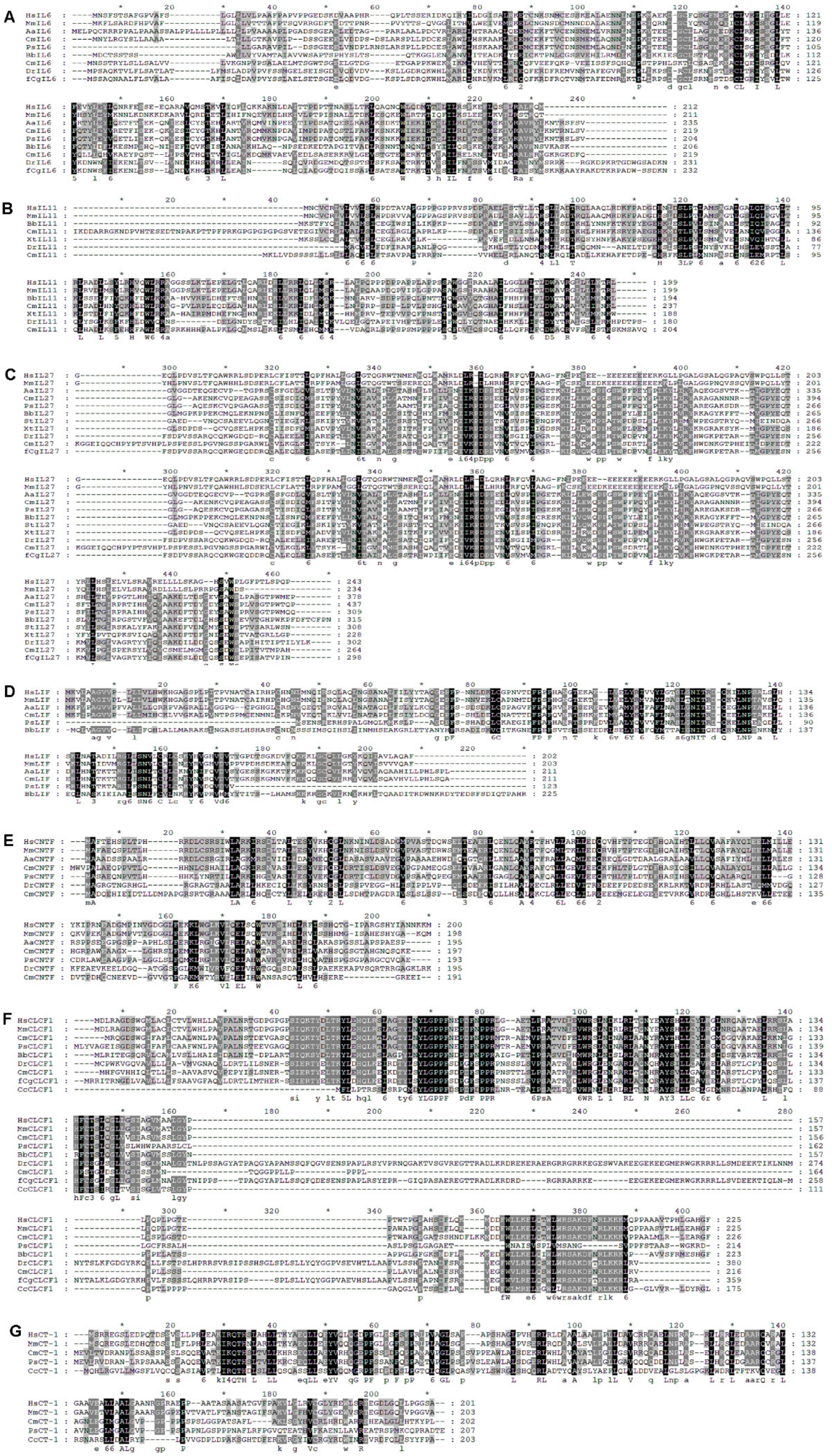
A
B
C
D
E
F
G

Figure S1 The sequence alignment of IL6 family members.

A. The sequence alignment of IL6s in species of metazoan phyla. *Hs*IL6, AAC41704.1; *Hs*IL6, AAC41704.1; *Mm*IL6, EDL37258.1; *Mm*IL6, NP_001248378.1; *Dr*IL6, NP_001248378.1; *Aa*IL6,— XP_051466182.1; *Cm*IL6, XP_043397018.1; *Ps*IL6, XP_006138413.1; *Bb*IL6, XP_040289612.1; *Om*IL6, NP_001118129.1; *fCg*IL6, XP_052441224.1. B. The sequence alignment of IL11s. *Hs*IL11, AAH12506.1; *Mm*IL11, AAC52941.1; *Dr*IL11, XP_021323960.1; *Om*IL11, CAI29480.1; *Xt*IL11, XP_031762208.1; *Bb*IL11, XP_040271531.1; *Cm*IL11, XP_037744116.2. C. The sequence alignment of IL27s. *Hs*IL27, KAI4054223.1; *Mm*IL27, EDL17402.1; *Dr*IL27, NP_001106980.1; *St*IL27, XP_048415378.1; *Aa*IL27, XP_051495931.1; *Cm*IL27, XP_027690128.2; *Ps*IL27, XP_025046796.1; *Bb*IL27, XP_040293780.1; *Xt*IL27, XP_017946498.1; *Om*IL27, NP_001117877.1; *fCg*IL27, XP_052403663.1. D. The sequence alignment of LIFs. *Hs*LIF, AAA51699.1; *Mm*LIF, EDL40470.1; *Aa*LIF, XP_051489984.1; *Cm*LIF, XP_007068602.1; *Ps*LIF, XP_006136764.1; *Bb*LIF, XP_040272631.1; *Om*LIF, XP_036835250.1. E. The sequence alignment of CNTFs. *Hs*CNTF, NP_000605.1; *Mm*CNTF, NP_740756.1; *Dr*CNTF, NP_001139104.1; *Aa*CNTF, XP_051477730.1; *Cm*CNTF, XP_037754603.1; *Ps*CNTF, XP_006120956.1; *Om*CNTF, NP_001165325.1. F. The sequence alignment of CLCF1s. *Hs*CLCF1, AAH66231.1; *Ms*CLCF1, NP_001365729.1; *Dr*CLCF1, XP_690733.6; CmCLCF1, XP_043405646.1; *Ps*CLCF1,–XP_025035099.1; *Bb*CLCF1, XP_040292768.1; *Om*CLCF1, CDQ61242.1; *fCg*CLCF1, XP_052470919.1; *Cc*CLCF1, XP_041029821.1. G. The sequence alignment of CT-1s. *Hs*CT-1, AAD12173.1; *Mm*CT-1, AAG21821.1; *Cm*CT-1, XP_037754619.1; *Ps*CT-1,-XP_025046100.1; *Cc*CT-1, XP_041038148.1.

Table S1. IL-6 family cytokines, their receptor complexes and their major biological roles

Cytokine	Receptor	Major biological roles
IL6	IL6R/ gp130	immune regulation, hematopoiesis, inflammation and oncogenesis[1]
IL11	IL11R/ gp130	anti-inflammatory effects [2], cell proliferation [3]
IL27	IL27R/ gp130	Th1 differentiation, suppression of Th2/Th17, antiviral immunity [4]
IL31	IL31R/ OSMR	Skin inflammation, pruritus[5], allergic responses[6]
OSM	OSMR/ gp130	Angiogenesis[7], bone remodeling, inflammation[8],
LIF	LIFR/ gp130	Tumor Progression[9], embryogenesis[10] haematopoiesis[11]
CNTF	CNTFR/ gp130	neuronal protection, energy metabolism [12]
CT-1	CRLF/ CNTFR	Angiogenesis [13], the survival of both cardiac and neuronal cells [14]
CLCF1	CRLF/ CNTFR	B-cell activation [15], kidney and lung pathology, osteoarthritis and haematopoiesis [16]

Table S2. List of species abbreviations

Abbreviation	Scientific Name	Taxonomic Group
Aa	*Apus apus*	Aves (Bird)
Ac	*Acropora cervicornis*	Cnidaria (Coral)
Aq	*Amphimedon queenslandica*	Porifera (Sponge)
Bb	*Bufo bufo*	Amphibia (Toad)
Bb	*Branchiostoma belcheri(Fig 5&7)*	Cephalochordata (Lancelet)
Bf	*Branchiostoma floridae*	Cephalochordata (Lancelet)
Bg	*Biomphalaria glabrata*	Mollusca (Gastropod)
Bl	*Branchiostoma lanceolatum*	Cephalochordata (Lancelet)
Bp	*Balaenoptera physalus*	Mammalia (Whale)
Ca	*Crassostrea angulate*	Mollusca (Bivalve)
Cc	*Carcharodon carcharias*	Chondrichthyes (Shark)
Cg	*Crassostrea gigas*	Mollusca (Bivalve)
Ci	*Ciona intestinalis*	Urochordata (Tunicate)
Cm	*Chelonia mydas*	Reptilia (Turtle)
Dm	*Drosophila melanogaster*	Arthropoda (Insect)
Dr	*Danio rerio*	Actinopterygii (Zebrafish)
Ef	*Ephydatia fluviatilis*	Porifera (Sponge)
Es	*Eriocheir sinensis*	Arthropoda (Crab)
fCg	*Carassius gibelio*	Actinopterygii (Gibel carp)
Gb	*Geodia barretti*	Porifera (Sponge)
Hr	*Haliotis rufescens*	Mollusca (Gastropod)

Hs	*Homo sapiens*	Mammalia (Human)
La	*Lingula anatine*	Brachiopoda (Lamp shell)
Lr	*Lethenteron reissneri*	Cyclostomata (Lamprey)
Mc	*Mytilus californianus*	Mollusca (Bivalve)
Me	*Mytilus edulis*	Mollusca (Bivalve)
Mg	*Mytilus galloprovincialis*	Mollusca (Bivalve)
Mm	*Mus musculus*	Mammalia (Mouse)
My	*Mizuhopecten yessoensis*	Mollusca (Bivalve)
Ob	*Octopus bimaculoides*	Mollusca (Cephalopod)
Oe	*Ostrea edulis*	Mollusca (Bivalve)
Of	*Owenia fusiformis*	Annelida (Polychaete)
Om	*Oncorhynchus mykiss*	Actinopterygii (Rainbow trout)
Os	*Octopus sinensis*	Mollusca (Cephalopod)
Pj	*Penaeus japonicus*	Arthropoda (Shrimp)
Pm	*Petromyzon marinus*	Cyclostomata (Lamprey)
Po	*Penaeus vannamei*	Arthropoda (Shrimp)
Ps	*Pelodiscus sinensis*	Reptilia (Turtle)
Pv	*Penaeus vannamei*	Arthropoda (Shrimp)
Sc	*Styela clava*	Urochordata (Tunicate)
Se	*Saccostrea echinata*	Mollusca (Bivalve)
Sk	*Saccoglossus kowalevskii*	Hemichordata (Acorn worm)
Sm	*Spheniscus magellanicus*	Aves (Penguin)
Sp	*Strongylocentrotus purpuratus*	Echinodermata (Sea urchin)
St	*Stegostoma tigrinum*	Chondrichthyes (Shark)
Xt	*Xenopus tropicalis*	Amphibia (Frog)

1. Kishimoto, T. IL-6: from its discovery to clinical applications. *Int Immunol* **2010**, *22*, 347-352, doi:10.1093/intimm/dxq030.
2. Ernst, M.; Putoczki, T.L. Molecular pathways: IL11 as a tumor-promoting cytokine-translational implications for cancers. *Clin Cancer Res* **2014**, *20*, 5579-5588, doi:10.1158/1078-0432.Ccr-13-2492.
3. Leng, S.X.; Elias, J.A. Interleukin-11. *Int J Biochem Cell Biol* **1997**, *29*, 1059-1062, doi:10.1016/s1357-2725(97)00017-4.
4. Yoshida, H.; Hunter, C.A. The immunobiology of interleukin-27. *Annu Rev Immunol* **2015**, *33*, 417-443, doi:10.1146/annurev-immunol-032414-112134.
5. Fassett, M.S.; Braz, J.M.; Castellanos, C.A.; Salvatierra, J.J.; Sadeghi, M.; Yu, X.; Schroeder, A.W.; Caston, J.; Munoz-Sandoval, P.; Roy, S., et al. IL-31-dependent neurogenic inflammation restrains cutaneous type 2 immune response in allergic dermatitis. *Sci Immunol* **2023**, *8*, eabi6887, doi:10.1126/sciimmunol.abi6887.
6. Patrick, M.T.; Wu, Y.; Zhong, X.; Li, Q.; Julia, V.; Li, B.; Gudjonsson, J.E.; Tsoi, L.C. IL31 identified as a key genetic risk factor for prurigo nodularis. *J Allergy Clin Immunol* **2025**, *156*, 1410-1413, doi:10.1016/j.jaci.2025.06.031.
7. Zhang, X.; Zhu, D.; Wei, L.; Zhao, Z.; Qi, X.; Li, Z.; Sun, D. OSM Enhances Angiogenesis

and Improves Cardiac Function after Myocardial Infarction. *Biomed Res Int* **2015**, *2015*, 317905, doi:10.1155/2015/317905.
8. Wolf, C.L.; Pruett, C.; Lighter, D.; Jorcyk, C.L. The clinical relevance of OSM in inflammatory diseases: a comprehensive review. *Front Immunol* **2023**, *14*, 1239732, doi:10.3389/fimmu.2023.1239732.
9. Shao, Y.; Lu, D.; Jin, W.; Chen, S.; Han, L.; Wang, T.; Fu, L.; Yu, H. Targeting LIF With Cyclovirobuxine D to Suppress Tumor Progression via LIF/p38MAPK/p62-Modulated Mitophagy in Hepatocellular Carcinoma. *MedComm (2020)* **2025**, *6*, e70227, doi:10.1002/mco2.70227.
10. Fry, R.C. The effect of leukaemia inhibitory factor (LIF) on embryogenesis. *Reprod Fertil Dev* **1992**, *4*, 449-458, doi:10.1071/rd9920449.
11. Kurzrock, R.; Estrov, Z.; Wetzler, M.; Gutterman, J.U.; Talpaz, M. LIF: not just a leukemia inhibitory factor. *Endocr Rev* **1991**, *12*, 208-217, doi:10.1210/edrv-12-3-208.
12. Guo, H.; Chen, P.; Luo, R.; Zhang, Y.; Xu, X.; Gou, X. The Roles of Ciliary Neurotrophic Factor - from Neuronutrition to Energy Metabolism. *Protein Pept Lett* **2022**, *29*, 815-828, doi:10.2174/0929866529666220905105800.
13. Zheng, Z.Z.; Tian Fu, X.; Liang, J.; Bing Guo, Z. CT-1 induces angiogenesis by regulating the ADMA/DDAH Pathway. *Biomed Pap Med Fac Univ Palacky Olomouc Czech Repub* **2015**, *159*, 540-546, doi:10.5507/bp.2015.009.
14. Latchman, D.S. Cardiotrophin-1 (CT-1): a novel hypertrophic and cardioprotective agent. *Int J Exp Pathol* **1999**, *80*, 189-196, doi:10.1046/j.1365-2613.1999.00114.x.
15. Savin, V.J.; Sharma, M.; Zhou, J.; Gennochi, D.; Fields, T.; Sharma, R.; McCarthy, E.T.; Srivastava, T.; Domen, J.; Tormo, A., et al. Renal and Hematological Effects of CLCF-1, a B-Cell-Stimulating Cytokine of the IL-6 Family. *J Immunol Res* **2015**, *2015*, 714964, doi:10.1155/2015/714964.
16. Sims, N.A. Cardiotrophin-like cytokine factor 1 (CLCF1) and neuropoietin (NP) signalling and their roles in development, adulthood, cancer and degenerative disorders. *Cytokine Growth Factor Rev* **2015**, *26*, 517-522, doi:10.1016/j.cytogfr.2015.07.014.
